# Determinants of cardiac troponin T elevation in COPD exacerbation – a cross-sectional study

**DOI:** 10.1186/1471-2466-9-35

**Published:** 2009-07-19

**Authors:** Pål H Brekke, Torbjørn Omland, Stein Harald Holmedal, Pål Smith, Vidar Søyseth

**Affiliations:** 1 Department of Medicine and Faculty Division, Akershus University Hospital, N-1478 Lørenskog, Norway; 2 Department of Radiology, Akershus University Hospital, N-1478 Lørenskog, Norway

## Abstract

**Background:**

Cardiac Troponin T (cTnT) elevation during exacerbations of chronic obstructive pulmonary disease (COPD) is associated with increased mortality the first year after hospital discharge. The factors associated with cTnT elevation in COPD are not known.

**Methods:**

From our hospital's database, all patients admitted with COPD exacerbation in 2000–03 were identified. 441 had measurement of cTnT performed. Levels of cTnT ≥ 0.04 μg/l were considered elevated. Clinical and historical data were retrieved from patient records, hospital and laboratory databases. Odds ratios for cTnT elevation were calculated using logistic regression.

**Results:**

120 patients (27%) had elevated cTnT levels. The covariates independently associated with elevated cTnT were increasing neutrophil count, creatinine concentration, heart rate and Cardiac Infarction Injury Score (CIIS), and decreasing hemoglobin concentration. The adjusted odds ratios (95% confidence intervals in parentheses) for cTnT elevation were 1.52 (1.20–1.94) for a 5 × 10^6^/ml increase in neutrophils, 1.21 (1.12–1.32) for a 10 μmol/l increase in creatinine, 0.80 (0.69–0.92) for a 1 mg/dl increase in hemoglobin, 1.24 (1.09–1.42) for a 10 beats/minute increase in heart rate and 1.44 (1.15–1.82) for a 10 point increase in CIIS.

**Conclusion:**

Multiple factors are associated with cTnT elevation, probably reflecting the wide panorama of comorbid conditions typically seen in COPD. The positive association between neutrophils and cTnT elevation is compatible with the concept that an exaggerated inflammatory response in COPD exacerbation may predispose for myocardial injury.

## Background

Chronic Obstructive Pulmonary Disease (COPD) constitutes an increasing health burden worldwide, and is the only leading cause of death that still has a rising mortality rate in industrialized countries.[1] In addition to a slow progression of symptoms and deterioration of lung function, a large proportion of the patients experience episodes of symptom worsening, including increased sputum, cough and dyspnea (exacerbations).[2] During exacerbations, patients frequently need hospitalization, and mortality is increased. Among COPD patients, cardiovascular comorbidities are common, with congestive heart failure and ischemic heart disease estimated to account for 10–40% of deaths.[3,4]

Cardiac troponins are established markers of myocardial damage, and were included in the diagnostic criteria for myocardial infarction in 2000.[5] Additionally, cardiac troponin elevation is also seen in a variety of conditions not directly related to flow-limiting coronary stenoses or occlusion of the coronary arteries, such as pulmonary embolism, septic shock, heart failure and stroke. [6-9] In these settings, it is well documented that elevated circulating levels of troponins are associated with poor prognosis, regardless of underlying disease.

In a historic cohort study of patients hospitalized for COPD exacerbation, we found that patients with cardiac troponin T (cTnT) ≥ 0.04 μg/l had a mortality rate nearly twice as high as patients with no measurable cTnT in the first year after discharge. [10]

With the exception of one other study,[11] sparse information exists concerning the significance of troponin elevation in COPD. Knowledge of determinants of troponin elevation in COPD exacerbation may improve our understanding of underlying pathophysiological processes, and ultimately, lead to improved therapeutic strategies. Accordingly, the aim of this study is to identify the clinical, biochemical and radiographic variables associated with cTnT elevation during admission to hospital for COPD exacerbation.

## Methods

The study population consisted of patients admitted to Akershus University Hospital, a 700-bed teaching hospital that serves suburban and countryside regions, for COPD exacerbation. Cases were identified using the hospital's patient database. Patients aged 40 years or older admitted during the four year period 1 January 2000 – 31 December 2003 and discharged with a primary diagnosis of COPD exacerbation, ICD 10 (International Classification of Diseases, 10th revision) code J44.0 or J44.1, or COPD (J44.x) as an underlying diagnosis combined with pneumonia (J13–J18.9) as the main diagnosis, were identified. Patients with previous diagnoses of asthma, sarcoidosis, interstitial lung disease, or neuromuscular disease were excluded. For patients with more than one admission during the inclusion period, the latest admission date was used. 1087 admissions satisfied inclusion and exclusion criteria. 41 patient records were not available for manual review. 50 cases of erroneous ICD coding (5%) were discovered during manual record review.

Of the 996 patients identified, we included the 441 patients from whom cTnT was sampled within the first 24 hours of admission in the current investigation. The decision to sample cTnT was made in the emergency room (ER) at the discretion of the physician on call, and was thus non-random. This selection process has been analysed and discussed in a previous paper where we used a propensity score model to adjust for possible sampling bias. [10]

Results from the first two rounds of laboratory analyses performed within 24 hours from the time of hospital admission were retrieved from the hospital's laboratory database, 86% of results were from the first set of blood samples obtained at presentation, before treatment was instigated. For white blood cell count (WBC), results from the first round of samples were used. Otherwise, when multiple results were available, average hemoglobin concentrations (Hb), maximum cTnT and C-reactive protein (CRP), and minimum creatinine values were used. WBC was divided into neutrophil count and non-neutrophil count.

The cTnT assay used by the hospital laboratory was Elecsys Troponin T STAT (Roche Diagnostics GmbH, Mannheim, Germany), the serum creatinine assay was an Ortho Vitros Crea enzymatic slide method, range 4–1238 μmol/L, CV 2.0% at 70 μmol/L (Ortho Clinical Diagnostics, Rochester, NY), C-reactive protein was analysed using a particle-enhanced turbidimetric immunoassay with a CV of 2–4% in the range of 0–300 mg/L, upper reference: 7 mg/L (Roche Diagnostics GmbH, Mannheim, Germany). Hematology analysis was performed on a Sysmex XE 2100 (Sysmex Europe GmbH, Norderstedt, Germany). Anemia was defined as Hb concentration < 12 mg/dl if female, < 13 mg/dl if male.

cTnT was considered elevated at levels equal to or greater than 0.04 μg/l, at which point the assay has less than 10% coefficient of variation (personal communication with our hospital laboratory), which is the cut-off criterium recommended by the ESC/ACC.[5] Hospital policy at that time recommended a cTnT concentration of 0.10 μg/l or higher as the diagnostic threshold for myocardial infarction (MI). The detection limit for cTnT was 0.01 μg/l.

Patient data from ER presentation, including oxygenation measured by pulse oximetry, supplemental oxygen flow, arterial blood gas analysis (pO_2_, pCO_2_, pH), blood pressure, and respiratory frequency, in addition to medication use on admission and at discharge, were manually gathered from hospital records.

ECGs recorded on admission were retrieved from patients' records, and manually scored according to a Cardiac Infarction Injury Score (CIIS) algorithm modified for visual coding.[12] Heart rate and rhythm were gathered from the ECG. Coders were blinded to other clinical data.

Spirometry data, if performed in a stable state at least one week before or four weeks after hospital discharge, were gathered from patient records. Forced vital capacity (FVC), expiratory volume in one second (FEV_1_) are expressed as percent of predicted using the European Community for Steel and Coal equations.[13]

Available chest radiographs from the date of admission were re-examined in co-operation by two physicians (VS and SHH), who were blinded regarding clinical data. The following parameters were evaluated: Cardiomegaly (cardiothoracic ratio > 50%), hyperinflation, pneumonic infiltrates, and pulmonary congestion.

Discharge ICD codes entered for each patient since 1987, until but not including the date of admission, were obtained from the hospital database. For each patient, groups of codes were used to construct a history of cancer, diabetes, hypertension, MI, congestive heart failure and venous thromboembolism. Additionally, each patient's medical records were manually searched for information on comorbid conditions.

The study was approved by The Data Inspectorate and the Regional Committee for Research Ethics.

### Statistical analyses

Chi-square test were used for between-group comparisons in univariate analyses on dichotomous variables (table 1), and univariate logistic regression was used for continuous variables. For descriptive purposes, continuous variables are shown as quartiles in table 2. In all regression models, however, these variables were used in their original form.

**Table 1:** Dichotomous variables as number of subjects, n (%), with elevated vs non-elevated cTnT, with p-values from chi square tests.

*Characteristic*	*cTnT *= *0.04* *(n≥ 120)*	*cTnT < 0.04* *(n = 321)*	*p-value*
**Gender**			
Female	51 (43)	166 (52)	0.09
			
**ECG**			
Atrial fibrillation	20 (17)	34 (11)	0.08
			
**Chest radiograph**			
Hyperinflation	31 (26)	99 (31)	0.31
Infiltrate	50 (43)	77 (27)	0.001
Cardiomegaly	69 (58)	111 (35)	< 0.001
Congestion (any sign)	53 (44)	94 (29)	0.003
			
**Medical history**			
Myocardial infarction	35 (33)	58 (19)	0.002
Heart failure	31 (26)	27 (8)	< 0.001
Diabetes	29 (24)	41 (13)	0.004
Hypertension	32 (27)	75 (23)	0.47
Thromboembolism	4 (3)	11 (3)	0.96
Cancer	17 (14)	41 (13)	0.7
			
**Treatment**			
LTOT	9 (8)	27 (9)	0.86
Inhaled beta_2_-agonists	91 (86)	250 (88)	0.62
Inhaled corticosteroids	60 (55)	202 (66)	0.04
Warfarin	16 (15)	33 (11)	0.30
Aspirin	45 (38)	93 (29)	0.09
ACE inhibitors	32 (29)	63 (21)	0.07
Beta-blockers	18 (16)	52 (17)	0.88
Statins	15 (13)	55 (17)	0.24

ACE – Angiotensin converting enzyme; ECG – Electrocardiogram; LTOT – Long term oxygen therapy

The multiple logistic regression model was built according to a purposeful variable selection method outlined by Hosmer and Lemeshow. [14] Briefly, all variables that were associated with cTnT elevation by univariate tests with a p-value equal to or less than 0.20 were included in the initial multivariate logistic regression model. This was then manually simplified by removing clearly non-significant covariates while for each step comparing the simpler model to the larger one using the likelihood ratio chi-square test and ensuring that variable removal did not meaningfully change the estimates for other important covariates. Continuous variables were tested for linearity in the logit by using design variables. All variables not initially included were tested for significance in the preliminary main effects model before testing for clinically interesting interactions. Finally, goodness of fit of the model was assessed using the Hosmer-Lemeshow test. Results of the regression are reported as odds ratios (OR), with 95% confidence intervals (CI) in parentheses.

## Results

The mean age of patients included in the study was 72.2 years (standard deviation 10.7), 49% were female. Elevated cTnT was found in 27% of patients. Coexisting cardiovascular conditions were common, with 24% of the patients having a diagnosis of arterial hypertension, 22% having a history of previous MI, 16% having diabetes mellitus, and 13% having a diagnosis of congestive heart failure. Spirometry data were available in 333 (76%) of patients. Mean FEV_1 _(pre-bronchodilator) was 48% of predicted, and mean FEV_1_/FVC-ratio was 0.67.

Univariate analyses identified clinical, laboratory and demographic variables associated with elevated cTnT levels, and results are presented in tables 1 and 2. Several markers of infection or inflammation (CRP, neutrophil count, infiltrate) were strongly associated with cTnT elevation, as were markers of heart disease (CIIS, radiographic cardiomegaly, radiographic signs of congestion, history of MI, heart failure or diabetes) as well as use of some drugs targeting the cardiovascular system (aspirin and angiotensin converting enzyme inhibitors) and inhaled corticosteroids. Use of beta-blockers or inhaled beta-agonists were not significantly associated with cTnT elevation, nor were history of hypertension, previous thromboembolic disease, pH, body temperature or non-neutrophil count.

**Table 2:** Continuous variables as number of subjects per quartile with elevated cTnT.

	*Quartiles*				
		
*Characteristic*	*First*	*Second*	*Third*	*Fourth*	*p*
**Age, years**	*< 64*	*64–73*	*74–80*	*> 80*	
n (%)	18 (17)	27 (25)	37 (32)	38 (34)	< 0.001
**Heart rate, beats/minute**	< 84	84–97	98–110	> 110	
n (%)	16 (15)	29 (29)	31 (29)	40 (40)	0.001
**CIIS, points**	< 6	6–13	14–22	> 22	
n (%)	17 (15)	22 (23)	34 (36)	43 (40)	< 0.001
**Systolic blood pressure, mmHg**	*< 120*	*120–139*	*140–157*	*> 157*	
n (%)	45 (41)	24 (24)	20 (21)	22 (20)	0.001
**Diastolic blood pressure, mmHg**	*< 65*	*65–76*	*77–88*	*> 88*	
n (%)	44 (37)	26 (27)	14(15)	26 (25)	0.002
**Temperature, °C**	*< 36.7*	*36.7–37.4*	*37.5–38.1*	*> 38.1*	
n (%)	26 (22)	37 (35)	18 (23)	27 (27)	0.9
**Respiratory rate, breaths/min**	*< 21*	*21–25*	*26–30*	*> 30*	
n (%)	10 (26)	11 (27)	9 (17)	20 (40)	0.04
**Oxygen saturation, %**	*< 88*	*88–92*	*93–95*	*>95*	
n (%)	25 (32)	18 (25)	21 (22)	20 (29)	0.06
**pCO_2_, kPa**	*< 4.8*	*4.8–5.5*	*5.6–6.7*	*> 6.7*	
n (%)	27 (26)	22 (22)	21 (24)	32 (36)	0.04
**pH**	*< 7.38*	*7.38–7.42*	*7.43–7.46*	*> 7.46*	
n (%)	42 (36)	13 (17)	26 (23)	21 (30)	0.10
**FEV_1_, % of predicted**	*< 31*	*31–42*	*43–58*	*> 58*	
n (%)	24 (33)	26 (33)	22 (26)	19 (20)	0.06
**Creatinine, μmol/l**	*< 62*	*62–76*	*77–97*	*> 97*	
n (%)	19 (18)	16 (16)	29 (30)	56 (44)	< 0.001
**CRP, mg/l**	*< 13*	*13–56*	*57–163*	*> 163*	
n (%)	21 (20)	24 (24)	27 (24)	47 (43)	0.001
**Neutrophil count, 10^6^/ml**	*< 5.8*	*5.8–8.3*	*8.4–11.7*	*> 11.7*	
n (%)	14 (17)	26 (21)	30 (27)	50 (41)	< 0.001
**Non-neutrophil count, 10^6^/ml**	*< 1.5*	*1.5–2*	*2.1–2.8*	*> 2.8*	
n (%)	40 (34)	19 (26)	21 (23)	23 (26)	0.11
**Hemoglobin, mg/dl**	*< 11.8*	*11.8–13.1*	*13.2–14.2*	*> 14.2*	
n (%)	49 (40)	33 (31)	16 (13)	22 (24)	< 0.001

P-values from univariate logistic regression.

CIIS – Cardiac infarction injury score; CRP – C-reactive protein; FEV1 – Forced expiratory volume in 1 second

Since increased neutrophil count is associated with infection, we investigated the possibility of an interaction between neutrophil count and the presence of a pneumonic infiltrate on the chest radiograph. The product term added to the preliminary main effects model was non-significant (p = 0.95).

After manual reduction of the logistic regression model, the covariates with significant independent associations with elevated cTnT were increasing creatinine concentration, neutrophil count, heart rate and CIIS points, and decreasing Hb. Adjusted ORs with corresponding 95% CIs are presented in Table 3. Eight patients were diagnosed with acute MI during the index hospitalisation. Removing these patients from the analysis produced negligible changes in the estimates in the final model.

**Table 3:** Results of multivariate logistic regression model. Adjusted odds ratios (OR), with 95% confidence intervals (CI), for elevated cTnT according to patient characteristic.

	*Full final model* *(cTnT ≥ 0.04, n = 344)*		*Restricted final model* *(cTnT 0.04 – 0.09, n = 263)*	
*Characteristic*	*OR*	*95% CI*	*OR*	*95% CI*
Creatinine + 10 (μmol/l)	1.21	1.12 – 1.32	1.11	0.99 – 1.94
Hemoglobin +1 (mg/dl)	0.80	0.69 – 0.92	0.84	0.71 – 1.00
Neutrophil count + 5 (10^6^/ml)	1.52	1.20 – 1.94	1.48	1.13 – 1.94
Heart rate + 10 (beats/minute)	1.24	1.09 – 1.42	1.17	1.00 – 1.36
CIIS +10 (points)	1.45	1.15 – 1.82	1.25	0.95 – 1.64

Odds ratios are adjusted for all other variables in the table.

CIIS – Cardiac infarction injury score.

The final model satisfied the Hosmer-Lemeshow test for goodness of fit, but exploring model diagnostics and coefficients for stratified variables revealed that the effect of heart rate was not entirely linear. While a better fitting model for heart rate data was achieved when eliminating patients with atrial fibrillation from the regression, this reduced model caused only marginal changes in the effect estimates for covariates at the third significant digit, with the exception of neutrophil count (OR: 1.42 (1.10–1.83) for a 5 × 10^6^/ml increase). Thus, the model presented in Table 3 is for the complete cohort. Neither age, gender nor pulmonary function were significantly associated with cTnT elevation in the multivariate model.

Due to the historic nature of the data, it is difficult to assess whether cTnT elevations represent an acute MI or not. However, by restricting the final model to patients with only marginally elevated cTnT concentrations, above the detection limit of 0.04 μg/L but below a commonly used MI decision level of 0.10 μg/L, most if not all patients with an acute MI should be excluded. This restricted model, while having less statistical power and rendering several covariates marginally significant, resulted in limited changes in the effect estimates. Neutrophil count remained highly significant in the restricted model.

## Discussion

The important new information derived from the present study is that multiple, apparently unrelated factors are associated with increased cTnT levels in patients hospitalized for COPD exacerbation. Accordingly, factors believed to signify coronary artery disease, tachycardia, renal impairment and anemia all contribute to cTnT elevation. Interestingly, neutrophils, which are the cells that characterize COPD exacerbations, were positively associated with cTnT elevation in a dose-response manner. Given that cTnT is a strong, independent predictor of adverse outcome in patients with COPD exacerbation,[10] these results suggest that cTnT levels reflect and integrate prognostic information from different, harmful pathophysiological processes.

In human myocardium, cTnT is mostly bound to the myofibrils, but approximately 6% is found in solution in the cytosol. Traditionally, troponins have been considered markers of myocardial cell necrosis. However, several studies have shown a biphasic or rapid rise and fall of troponins in response to short term myocardial ischemia, suggesting that some of the circulating troponins may stem from reversible cell damage, probably representing the cytosolic fraction.[15,16] The restricted version of the final model, which includes only patients with a cTnT concentration highly unlikely to represent an acute MI, did not differ appreciably from the full model in effects estimates, but the lower statistical power rendered most covariates marginally significant. Neutrophil count remained highly significant. While we do not have follow-up troponin measurements from later in the hospital stay, the authors believe it is unlikely that the low levels of circulating cTnT observed in the present study reflect major ischemic events or occlusion of significant coronary arteries. Rather, our results suggest they may result from the total burden of multiple comorbidities in addition to the circulatory and respiratory strain of the COPD exacerbation itself.

There are multiple mechanisms which may account for the cTnT elevations observed in the present study. In the setting of a COPD exacerbation, arterial hypoxemia, tachycardia as well as anemia may cause a supply-demand mismatch in susceptible tissues, resulting in cardiomyocyte injury. These mechanisms may be applicable in patients with established coronary artery disease.[17] Perhaps surprisingly, our multivariate analyses did not confirm any significant association between arterial hypoxemia and cTnT elevation. Patients may have had more severe hypoxemia prior to hospitalization. The associations with anemia and tachycardia, however, are in agreement with previous studies.[18,19] In a similar manner, our study confirms the association between cTnT elevation and renal dysfunction.[20,21]

The association with an elevated neutrophil count may deserve particular attention. COPD is characterized by pronounced neutrophil inflammation of the airways, and during disease exacerbations there is a flaring of the inflammatory response, both locally and systemically.[22,23] A "spill-over" effect from the lungs to the systemic and cardiac vasculature is conceivable, possibly amplifying the inflammatory processes in atherosclerosis and atherothrombosis.[4,24] Interestingly, there was no sign of interaction between neutrophil inflammation and pneumonic infiltrates, suggesting that the mechanism is independent of infection. Indeed, increased circulating neutrophils have been implicated in myocardial damage through several mechanisms – including the secretion of large amounts of inflammatory mediators, activation of the coagulation system, oxidative vascular damage or even abnormal aggregation and vessel plugging.[25] Since steroid therapy may influence the WBC, we used only analysis results from the initial ER presentation, when blood samples are routinely drawn before therapy is started. Some patients may, however, have started treatment of the exacerbation prior to hospitalization and thus have an 'artificially' increased WBC. This we have no way to control for, and the direction of bias is hard to determine: If patients with more severe disease are more likely to start steroids prior to hospitalisation, and have a higher risk of cTnT elevation, it would tend to strengthen the observed relationship between cTnT and WBC. On the other hand, studies on steroid use in COPD suggest a cardioprotective effect,[26,27] and pre-hospital steroid use would then tend to obscure the association between WBC and elevated cTnT, meaning that the true association may be stronger than our estimates.

The CIIS system was developed to improve the diagnosis of MI, and at score of 20 points has a sensitivity and specificity for detecting MI of 85% and 95%, respectively.[28] CIIS has been shown to be the best of several ECG scoring systems for determining risk of cardiovascular mortality [29] – additionally, the score has been shown to be inversely correlated with FEV_1_,[30] and carries independent prognostic information in COPD.[31] In the present study, we interpret CIIS as a better marker of established coronary artery disease in COPD patients than ICD diagnostic codes or history from medical records. In the final multivariate regression analysis, the inclusion of CIIS rendered the other covariates associated with a history of cardiovascular disease completely insignificant.

In our material, 27% of patients had elevated circulating cTnT. This is very close to the 25% prevalence found by Harvey and Hancox,[32] and comparable to the study by Baillard et al where they reported that 18% of their patients had elevated troponin I.[11] However, since cTnT in our study was measured in less than half of patients with COPD exacerbation, a selection of patients with an increased likelihood of troponin elevation is likely, thus resulting in our estimate of the prevalence of cTnT elevation probably being an overestimate of the true prevalence among patients hospitalized for COPD exacerbation. We have previously shown in a propensity score model on the same dataset that patients with reduced FEV_1 _or hyperinflation on chest radiograph had a lower probability of cTnT sampling, whereas a history of ischemic heart disease, use of aspirin, or radiographic signs of pulmonary congestion increased the probability of cTnT measurement. Mortality, however, was only marginally different between patients who did and did not have cTnT sampled.[10] This illustrates the difficulty in distinguising between COPD patients with higher and lower risks of myocardial damage in clinical practice, and indirectly supports the external validity of the present results.

The major methodological limitation of the present study is the cross-sectional design, which does not permit causal inferences to be made. Additionally, the historic nature of the data means that several interesting measurements possibly associated with cTnT elevation are unavailable, such as B-type Natriuretic Peptide or echocardiographic data for better classification of heart failure, or CT scans to diagnose pulmonary embolism. Finally, whether cTnT elevation in COPD exacerbations reflects an acute MI or is caused by other factors is difficult to establish without coronary imaging. The authors believe, however, that we have been able to account for significant factors using proxy variables and restricted analyses.

The diagnosis of COPD in this study was made at discharge, based on all available clinical data. Frequently, deciding whether or not COPD exacerbations are accompanied by pneumonia is difficult. Thus, we included patients coded with pneumonia as the main diagnosis and COPD as the underlying diagnosis. Testing for interaction revealed no significant difference in determinants of cTnT elevation for patients with pneumonic infiltrates compared to those without. A physician specialized in internal medicine or pulmonary medicine verified the diagnoses at the time of hospital discharge. While the diagnosis was not based on specific criteria such as suggested by the Global initiative for chronic Obstructive Lung Disease (GOLD), we consider the study population to be well defined, and representative of COPD patients seen in pulmonary care units in Western countries [33].

### Clinical implications

Given the prognostic implications of cTnT elevation, further diagnostic and therapeutic measures directed toward patients presenting with risk factors for cTnT elevation may be warranted. Intensified surveillance and treatment of anemia and tachycardia, and increased diagnostic and secondary prevention efforts in patients with markers of coronary artery disease could potentially reduce the incidence of troponin elevation in COPD exacerbation. However, considering the multiple factors involved in cTnT elevation, interpretation of low grade cTnT elevation in a COPD population should be done with caution. Elevated cTnT in isolation should not be considered diagnostic of an acute MI, or an indication for angiography. That said, the angiographic correlates of cTnT elevation in COPD remain to be elucidated.

As to the neutrophil count, given that systemic and cardiac inflammatory responses are caused or exacerbated by the local inflammation associated with COPD, there is a possibility of mitigating that response through local treatment. At least two papers have shown that inhaled corticosteroid use in COPD causes reduction in systemic markers of inflammation.[34,35] Most intriguingly, a study of therapy with corticosteroids by Huiart et al indicated a 32% reduction in the risk of acute MI (p < 0.05) for patients using 50–200 μg per day of inhaled beclomethasone or equivalent,[26] and a *post hoc *analysis of the European Respiratory Society's study on Chronic Obstructive Pulmonary Disease (EUROSCOP) trial showed that smokers with mild COPD treated with inhaled budesonide 800 μg/day had a significantly lower incidence of ischemic cardiac events compared to patients receiving placebo.[27]

## Conclusion

The present study suggests a multifactorial explanation for the cTnT elevation seen in COPD exacerbation, partly reflecting the many comorbid conditions seen in COPD. The positive association between neutrophils and cTnT elevation is compatible with the concept that an exaggerated inflammatory response in COPD exacerbation predisposes for myocardial injury.

## Competing interests

Torbjørn Omland has received speaker's honoraria and research support from Roche Diagnostics, manufacturer of the troponin T assay used in this analysis. None of the other authors have any conflicts of interest to disclose.

## Authors' contributions

PHB participated in the design of the study and the data gathering, did most of the statistical analyses and wrote the main draft of the manuscript. VS conceived of the study, participated in the design, data gathering, and statistical analyses and helped draft and revise the manuscript. SHH participated in the design and data gathering, and helped draft the manuscript. PS and TO participated in the design and coordination of the study, in the interpretation of data, and helped draft and revise the manuscript. All authors read and approved the final manuscript.

## Pre-publication history

The pre-publication history for this paper can be accessed here:


